# E‐cadherin is a robust prognostic biomarker in colorectal cancer and low expression is associated with sensitivity to inhibitors of topoisomerase, aurora, and HSP90 in preclinical models

**DOI:** 10.1002/1878-0261.13159

**Published:** 2021-12-26

**Authors:** Jarle Bruun, Peter W. Eide, Christian Holst Bergsland, Oscar Bruck, Aud Svindland, Mariliina Arjama, Katja Välimäki, Merete Bjørnslett, Marianne G. Guren, Olli Kallioniemi, Arild Nesbakken, Ragnhild A. Lothe, Teijo Pellinen

**Affiliations:** ^1^ Department of Molecular Oncology Institute for Cancer Research Oslo University Hospital Norway; ^2^ K.G. Jebsen Colorectal Cancer Research Centre Division for Cancer Medicine Oslo University Hospital Norway; ^3^ Hematology Research Unit Helsinki University of Helsinki and Comprehensive Cancer Center Helsinki University Hospital Finland; ^4^ Institute for Clinical Medicine Faculty of Medicine University of Oslo Norway; ^5^ Department of Pathology Oslo University Hospital Norway; ^6^ Institute for Molecular Medicine Finland HiLIFE University of Helsinki Finland; ^7^ Department of Oncology Oslo University Hospital Norway; ^8^ Science for Life Laboratory Department of Oncology & Pathology Karolinska Institutet Stockholm Sweden; ^9^ Department of Gastrointestinal Surgery Oslo University Hospital Norway

**Keywords:** colorectal cancer, drug screening, E‐cadherin, multiplex immunohistochemistry, pharmacoproteomics, prognostic biomarker

## Abstract

Cell–cell and cell–matrix adhesion proteins that have been implicated in colorectal epithelial integrity and epithelial‐to‐mesenchymal transition could be robust prognostic and potential predictive biomarkers for standard and novel therapies. We analyzed *in situ* protein expression of E‐cadherin (ECAD), integrin β4 (ITGB4), zonula occludens 1 (ZO‐1), and cytokeratins in a single‐hospital series of Norwegian patients with colorectal cancer (CRC) stages I–IV (*n* = 922) using multiplex fluorescence‐based immunohistochemistry (mfIHC) on tissue microarrays. Pharmacoproteomic associations were explored in 35 CRC cell lines annotated with drug sensitivity data on > 400 approved and investigational drugs. ECAD, ITGB4, and ZO‐1 were positively associated with survival, while cytokeratins were negatively associated with survival. Only ECAD showed independent prognostic value in multivariable Cox models. Clinical and molecular associations for ECAD were technically validated on a different mfIHC platform, and the prognostic value was validated in another Norwegian series (*n* = 798). In preclinical models, low and high ECAD expression differentially associated with sensitivity to topoisomerase, aurora, and HSP90 inhibitors, and EGFR inhibitors. E‐cadherin protein expression is a robust prognostic biomarker with potential clinical utility in CRC.

## Graphical abstract

Cell–cell and cell–matrix adhesion proteins have been implicated in colorectal cancer (CRC) progression. Here, we preformed multiplex immunohistochemistry analyses of E‐cadherin (ECAD), zona occludens‐1 (ZO‐1), integrin‐ß4 (ITGB4), and pan‐cytokeratins (PanCK) in two CRC patient series. We identified ECAD as a robust, independent, prognostic biomarker. Low expression of ECAD was associated with low differentiation grade, microsatellite instability, and *BRAF* mutations. In addition, pharmacoproteomic analyses of CRC cell lines demonstrated that ECAD levels modulated the sensitivity to EGFR, topoisomerase, aurora, and HSP90 inhibitors.

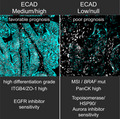

**Abbreviations**
5‐FU: 5‐fluorouracil
CI: confidence interval
CRC: colorectal cancer
DSS: drug sensitivity score
ECAD: E‐cadherin
EMT: epithelial‐to‐mesenchymal transition
FBS: fetal bovine serum
FDR: false discovery rate
HR: hazard ratio
ITGB4: integrin β4
mfIHC: multiplex fluorescence‐based immunohistochemistry
MMR: mismatch repair
MOA: mechanism of action
MSI: microsatellite instability
MSS: microsatellite stable
ND: not determined
NS: not significant
OS: overall survival
PanCK: pan‐cytokeratin
R0: complete resection
RFS: relapse‐free survival
TFT: test for trend
TMA: tissue microarray
TNM: tumor node metastasis
ZO‐1: zona occludens 1

## 1 Introduction

Epithelial cancers, the carcinomas, make up over 80% of cancer cases and deaths, where the majority of cancer patients die from metastatic disease [1]. Deregulation of epithelial cell adhesion junctions is an early part of the metastatic process and a prerequisite for cellular invasion and dissemination of cancer cells to neighboring tissues and organs and is therefore likely to be associated with patient survival. Carcinoma cells show phenotypic plasticity through differentiation programs such as epithelial‐to‐mesenchymal transition (EMT), where epithelial cells lose their apical‐to‐basal polarity and cell‐to‐cell junctional integrity by disassembly of tight junctions (zona occludens 1, ZO‐1), adherence junctions (E‐cadherin, ECAD), and hemidesmosomes (integrin beta 4, ITGB4, and cytokeratins) [2, 3]. For instance, loss or aberrant expression of ECAD is a hallmark of EMT, the transformation of cancer cells toward a mesenchymal stem cell‐like state which is associated with altered drug sensitivities [4].

Ten percent of all cancer cases and deaths are due to colorectal cancer (CRC) [5] and about half of the patients develop metastatic disease. The TNM staging system is used as the main framework to guide treatment, but prognosis and treatment efficacy vary considerably within cancer stages, and both overtreatment and undertreatment are challenges. Systemic treatment is primarily based on 5‐fluorouracil (5‐FU) in combination regimens with oxaliplatin or irinotecan or as monotherapy, while biologically targeted therapies are offered in the metastatic setting mainly with VEGF inhibition, or biomarker guided for tumors that are *KRAS/NRAS* wild‐type (anti‐EGFR therapy), have *BRAF* V600E mutation (BRAF inhibitor) [6] or are microsatellite instable (immune checkpoint therapy) [7]. However, for the majority of patients, few biomarkers exist to guide oncological intervention, either for systemic chemotherapy or for other targeted therapies such as anti‐VEGF therapy or treatment with the multikinase inhibitor regorafenib. Hence, there is an unmet need for clinically useful prognostic and predictive biomarkers for this purpose.

Despite large biomarker discovery efforts over the last four decades, very few biomarkers have been integrated in clinical practice [8] and only a small percentage of patients benefit from genome‐driven oncology [9]. The reasons for biomarker failure are many [10] and include lack of independent validation in representative patient series, insufficient reporting, and use of inadequate laboratory procedures.

**Fig. 1:**
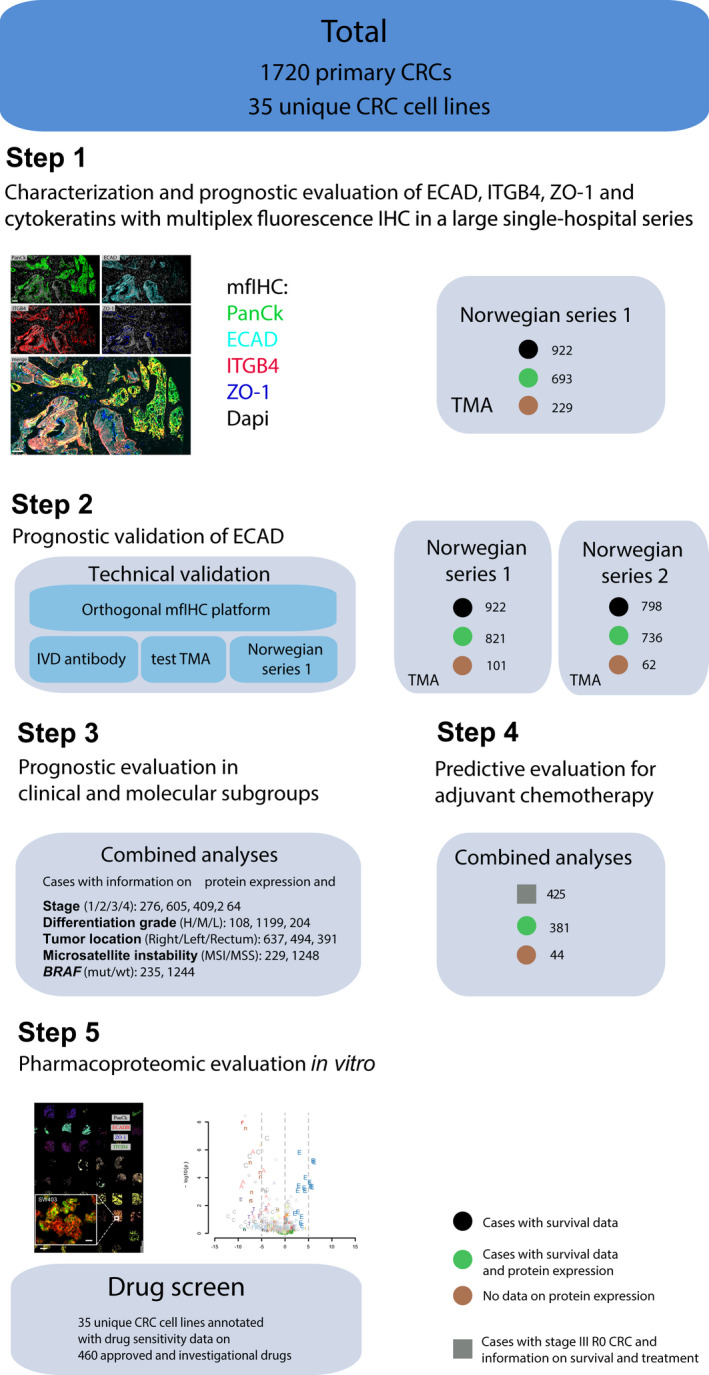
Study overview. Diagram showing patients and cell lines included and the analyses performed in the study. ECAD, E‐cadherin; ITGB4, integrin β4; IVD, *in vitro* diagnostic; MSI, microsatellite instable; MSS, microsatellite stable; R0, complete resection/no residual tumor; TMA, tissue microarray; ZO‐1, zona occludens‐1.

Loss of epithelial integrity and adhesion is necessary for metastasis, but systematic analysis of their implications for patient survival as well as tumor cell responses to conventional and novel oncologic therapies is lacking for CRC. We hypothesized that proteins necessary for colonic epithelial integrity could be robust prognostic biomarkers and predict efficacy of anticancer agents. The aim of this study was to explore and validate the clinical biomarker value of four protein markers implicated in cell–cell and cell–matrix adhesion using primary CRC‐resected tissue material (*n* = 1720) and to study their associations with drug response (*n* > 400 drugs) in 35 CRC cell lines (see Fig. 1 for the study overview).

## 2 Materials and methods

The design and methodology used in this study were reported according to the REMARK guidelines [11] (Table S1).

### 2.1 Patient samples

Two independent single‐hospital patient series of primary CRC were analyzed for epithelial marker expression using multiplex fluorescence‐based immunohistochemistry (mfIHC) (Fig. 1 and Table S2). Patients in the Norwegian series 1 (*n* = 922) and the Norwegian series 2 (*n* = 798) underwent major resection surgery at Oslo University Hospital, in the time periods 1993–2003 and 2003 to 2013, respectively. Clinical data were recorded in a local database and quality control was performed. The Cancer Registry of Norway records data on all patients diagnosed with CRC and was used to cross‐check the data used in this study. The series are representative of the Oslo area and considered of sufficient size to perform relevant subgroup analyses. Information on tumor location, histopathological grade, stage, adjuvant chemotherapy, deaths, and locoregional and/or distant recurrence during 5‐year regular follow‐up was registered prospectively.

Adjuvant chemotherapy was administered according to national guidelines at the given time periods. From 1997 adjuvant chemotherapy became standard treatment for all stage III colon cancer patients in Norway up to 75 years of age expected to tolerate such treatment. Adjuvant chemotherapy was given on a case‐by‐case basis according to risk assessments in stage III colon cancer patients above 75 years of age (*n* = 6/140) or with stage II or III rectal cancers (*n* = 10/260). Five‐year follow‐up for cancer recurrence and survival was complete for all patients except two (one censored at 4.2 years and one with missing information).

For the Norwegian series 1, DNA was extracted, MSI status was determined, and a tissue microarray (TMA) was constructed from matching formalin‐fixed paraffin‐embedded (FFPE) tumor tissue, as described in [12, 13]. For the Norwegian series 2, DNA was extracted from fresh‐frozen tumor tissue for a subset of the patients and MSI status was determined as previously described [14]. Matching FFPE tumor tissue was used to construct a TMA and additional data on MSI status were obtained from staining the Norwegian series 1 and 2 for MLH1, MSH2, MSH6, and PMS2 protein [15]. Sequencing of *BRAF* in exon 15 (including codon 600) was performed for a subset of the patients on a 3730 DNA Analyzer (Applied Biosystems, Foster City, CA, USA) as previously described [14]. Additional data on *BRAF* mutational status were obtained from IHC staining using the anti‐BRAF V600E (clone VE1) mouse monoclonal antibody from Ventana (Roche, Tucson, AZ, USA) according to the manufacturer’s instructions. For the two series combined, *BRAF* mutational status was available for 1114 patients and 1236 were scored by IHC (750 had overlapping data; the concordance between methods was 96%). In cases of discrepancy, the results from *BRAF* mutational analyses were used. The two series were merged for the final ECAD analyses to increase the statistical power of the multivariable models and subgroup analyses.

This project was approved by the Norwegian Data Protection Authority and the Regional Committee for Medical and Health Research Ethics, South‐Eastern Norway (REK number 1.2005.1629), and written informed consent was obtained from all patients prior to enrollment. The research was carried out according to the Declaration of Helsinki, and the research biobanks were constructed according to national legislation.

### 2.2 Cell culture

Cell lines were obtained from various providers as previously described in [16], and HT55, SW1417, and SW1222 were purchased from the European Collection of Authenticated Cell Cultures (ECACC). Identities of the cell lines were confirmed using short tandem repeat profiling with the AmpF‘STR Identifiler PCR Amplification Kit (Life Technologies by Thermo Fisher Scientific, Waltham, MA, USA). Cell lines were kept in DMEM/F12 (except from CaCo_2_ and WiDr cells which were kept in EMEM) supplemented with fetal bovine serum (FBS), 2 mm glutamine, 100 units·mL^−1^ penicillin, and 100 μg·mL^−1^ streptomycin (Gibco, Life Technologies, Carlsbad, CA, USA) and kept at 37 °C and 5% CO_2_ in a humidified incubator. Culture media was enriched with 10% FBS (20% for CaCo_2_ cells). The MycoAlert Mycoplasma Detection Assay (Lonza, Cologne, Germany) was used to regularly test for mycoplasma contamination.

### 2.3 Cell line paraffin arrays

Thirty‐five unique CRC cell lines were cultured and detached by trypsinization at exponential growth phase and fixed using Shandon fixative for 20 min (RT). Cell pellets were washed with Tris‐buffered saline (TBS) and processed to paraffin using Cytoblocks (Cat No 10066588; Thermo Scientific). Punches (1 mm core) with two replicates were taken from donor cell line paraffin blocks to generate the cell line paraffin array blocks.

### 2.4 Multiplex fluorescence‐based immunohistochemistry

Multiplex IHC protocols were conducted using 4–μm‐thick sections. The analyses did not include cases with poor tumor preservation, loss of tissue, low number of epithelial cells (typically < 50), extensive tissue folding, or necrosis.

#### 2.4.1 Norwegian series 1 and cell line paraffin arrays

For IHC staining of the Norwegian series 1, we used a protocol described in [17]. The primary antibodies and fluorescence detection reagents were the following: PanCK (C‐11, Abcam, Cambridge, UK, AB7753, 1 : 1500; AE1/3, InVitrogen, Carlsbad, CA, USA, MA5‐13156, 1 : 1000) with TSA‐488 detection (Life Technologies, Eugene, Oregon), ZO‐1 (1 : 500; CST, Danvers, MA, USA, D6L1E) with TSA‐555 detection, ITGB4 (1 : 100; CST, 14803S) with anti‐rabbit‐AF647 detection (Thermo Fisher Scientific), ECAD (1 : 500, BD 610182) with anti‐mouse‐AF750 detection (Abcam, AB175738). Slides were then stained with DAPI (Roche, 5 µg·mL^−1^), mounted with ProLong Gold (Thermo Fisher Scientific) and whole‐slide imaged (see imaging below).

### 2.5 Tissue and cell line imaging

#### 2.5.1 Norwegian series 1

Five‐channel fluorescent images were acquired using Metafer 5 scanning and imaging platform (MetaSystems, Altlussheim, Germany) consisting of AxioImager.Z2 (Zeiss, Oberkochen, Germany) microscope equipped with Zeiss Plan‐Apochromat 2× objective (NA 0.8), CoolCube 2m CCD camera (MetaSystems), PhotoFluor LM‐75 (89 North PhotoFluor LM‐75, Meyer Instruments, Inc., Houston, TX, USA) metal‐halide light source, and Zeiss EPLAX VP232‐2 power supply. DAPI, FITC, Cy3, Cy5, and Cy7 filters were used with the following exposure times: DAPI = 3.1 ms, FITC (PanCK) = 1.6 ms, Cy3 (ZO‐1) = 2.2 ms, Cy5 (ITGB4) = 50 ms, Cy7 (ECAD) = 400 ms. Nine field of views were acquired per each TMA spot, composed using VSlide (MetaSystems), and the images were exported as one tiled image per spot as Lossless compressed TIFFs (95% resolution) for image analysis.

### 2.6 Digital image analysis

#### 2.6.1 Norwegian series 1 and cell line paraffin array

The image analysis was carried out using a cell image analysis software (cellprofiler version 2.2.0) [18]. Epithelial marker expressions were measured as mean pixel intensities within epithelial areas of the tissue microarray spots. The pipeline consisted of (a) detection of spot by pixel thresholding (all channel pixels as maximum) and removing spot edges by convex hull + erode, (b) detection of epithelium by summing PanCK + ECAD channel pixels and thresholding using global manual threshold and closing + dilating the mask, (c) measurement of marker expressions in the epithelial mask (measure object intensity within epithelial objects), and (d) exportation of the marker intensity values as CSV data. Cell line arrays were analyzed as above, except the pipeline did not have step 2, detection of epithelium. Cell line array marker expression measurements were done within the area specified by the cell spot (all pixels maximum + threshold).

#### 2.6.2 Scoring of cell lines for epithelial markers

For the scoring of cell lines with intensity categories 0–2, we combined digital image analysis and visual scoring. A consensus score was formed between the digital image analysis and visual interpretation of the stains. This is due to the fact that cell paraffin arrays contained technical artifacts of unfocused and folded sample areas.

### 2.7 Technical and clinical validation of ECAD in the Norwegian patient series 1 and 2

#### 2.7.1 Multiplex fluorescence‐based IHC

For technical and clinical validation of the prognostic value of ECAD, we stained the Norwegian series 1 and 2 using mfIHC following the protocol described in [19]. Briefly, a 5‐plex stain was optimized according to the Opal™ Multiplex IHC method (PerkinElmer/Akoya, Menlo Park, CA, USA). The Dako PT link module was used to remove paraffin from the glass slides, for heat‐induced epitope retrieval and to strip the antibodies (20 min at 97 °C) with the EnVision™ FLEX Target Retrieval Solution (3‐in‐1) pH 9 (pH 6 for PTEN and for cytokeratins) (Agilent/Dako, Santa Clara, CA, USA), in 65 °C preheat mode. The multiplex staining protocol was conducted with the Opal™ 5‐Color Manual IHC Kit (PerkinElmer/Akoya) following the manufacturer’s recommendations. Tissue sections were incubated for 30 min with these primary antibodies: ECAD (1 : 200, clone NCH‐38, Dako/Agilent; detected by Opal 520 at 1 : 100), Ki67 (1 : 4, RTU clone MIB‐1, Dako/Agilent; detected by Opal 570 at 1 : 100), PTEN (1 : 125, clone D4.3, Cell Signaling Technology, Danvers, MA, USA; detected by Opal 620 at 1 : 100). A cocktail of epithelial markers was employed in the final cycle to facilitate epithelial segmentation during image analysis using anti‐pan cytokeratin (1 : 1500, clone C‐11, Abcam) and anti‐pan cytokeratin type I/II (1 : 1000, clone AE1/AE3, Thermo Fisher Scientific); detected by Opal 690 at 1 : 100. DAPI (PerkinElmer/Akoya) was used as counterstain prior to mounting using ProLong Diamond Antifade Mountant (Invitrogen/Thermo Fisher Scientific). Singleplex stains were made for all fluorophores in order to make spectral signatures to unmix the multiplex. The spectral signature for the tissue autofluorescence was made from a slide without fluorophore.

Optimal antibody concentrations were determined by evaluating the specificity, intensity, and signal to noise of the individual signals using both chromogenic DAB and fluorescence stains. A dedicated test TMA was used for this purpose, including 42 primary CRC cases and six cases from normal colon mucosa. Recommended signal ranges and balancing of the fluorescence signals were used for all markers. A negative control stain was made by excluding the primary antibody on one slide.

#### 2.7.2 Image acquisition and digital image analysis

Standard settings at 20× magnification were used to generate multispectral images using the Vectra 3.0 Automated Quantitative Pathology Imaging System, 200 slides (vectra software version 3, PerkinElmer/Akoya). Multispectral image analysis of multiplex IHC stains was performed using inform Image Analysis Software (version 2.3, Akoya Biosciences). For training of the image analysis algorithm, representative images were initially loaded and spectrally unmixed with the spectral libraries made based on the individual fluorophore library stains and the tissue autofluorescence slide. After that, a machine‐learning algorithm was trained by the investigator specifying relevant tissue annotations aided by the signal from the epithelial markers to accurately segment tumor tissue versus stromal tissue and background, as well as individual cells based on the nuclear DAPI signal. Review of all images was performed after batch processing. Protein expression was quantified as the mean signal intensity within the whole epithelial compartment.

### 2.8 Drug screen

The drug screen was performed as described in [16]. Briefly, cell lines were screened with two different libraries including in total 620 approved and investigational small‐molecule drugs at five different concentrations spanning a 10 000‐fold concentration range. Growth patterns and rates of all the cell lines were carefully assessed with regard to viability and morphology making sure the cells were in a logarithmic growth phase throughout the experiments. An Echo 550 (Labcyte Inc., Sunnyvale, CA, USA) was used to preprint the drugs on 384‐well optical microplates. A Multidrop Combi Reagent Dispenser (Thermo Fisher Scientific) was used for cell seeding and the cells were assessed for viability after 72 h with the CellTiter‐Glo (CTG) assay (Promega, Fitchburg, WI, USA). The resulting luminescence signals were measured using a PHERAstar FS microplate reader (BMG Labtech GmbH, Ortenberg, Germany). Drug measurements were compared and normalized to DMSO wells (0.1%, negative control) and benzethonium chloride wells (100 μm, positive control). Efficacy of the individual drugs was estimated as a drug sensitivity score (DSS) according to the model developed by [20]. Only drugs with maximum DSS > 10 were considered in the analyses (*n* = 293).

### 2.9 Statistics

SPSS version 21.0 (IBM Corporation, Armonk, NY, USA), STATA version 15.1 (StataCorp, Lakeway Drive College Station, TX, USA), and rstudio version 1.2. 5019 (r version 3.6.2) were used for the statistical analyses. Five‐year relapse‐free (RFS) and 5‐year overall survival (OS) plots were generated by the Kaplan–Meier method. The logrank test was used to compare survival curves, while the Cox proportional hazards model was used to calculate hazard ratios (HR) and confidence intervals (CI) for disease recurrence. RFS was defined as the time from surgery to the first event of either locoregional recurrence or metastasis, or death from any cause. Any second primary cancer or other cancer for the same patient was ignored. The RFS analyses included only patients with complete resection. The patients were censored at loss to follow‐up, defined as the last date for clinical or radiological examination or at five years after surgery.

The functional forms of individual protein expressions were evaluated with the mfp algorithm in Stata, and a log2 transformation was found to be an adequate representation. For ECAD, the raw protein expression was log2‐transformed and normalized as *Z*‐scores prior to combined downstream analyses for the two Norwegian series.

Multivariable model‐building was based on the fit of the data, the interpretability of the covariates and their consistency with subject‐matter knowledge, with the aim to facilitate replication by other researchers and transportability to other settings. Clinical relevance determined which parameters to evaluate in multivariable models. Tumor differentiation grade is associated with patient survival (Fig. S1) and was included in initial models and was also found to correlate with ECAD protein expression as expected but was not included in final models because it is difficult to reproduce robustly among pathologists. However, the models and the prognostic effect of ECAD were only slightly affected by including differentiation grade in initial multivariable models (Tables S3 and S4). Adjuvant chemotherapy, as well as pre‐ and post‐operative radiotherapy for rectal cancer patients, was also considered in initial multivariable models, but was relevant for relatively few patients and did not change the models. Small groups were avoided to increase the stability of multivariable models. Hence, mucinous tumors were grouped with poorly differentiated tumors due to their similar prognosis. Synchronous tumors were excluded from the analyses since they were few and the location of the tumor with evaluable ECAD protein expression uncertain. The independent prognostic effect of ECAD was evaluated and confirmed in both full multivariable models and stepwise models with backward elimination (*P* < 0.05 inclusion and *P* > 0.1 exclusion). Full multivariable models were chosen as the final models because they are simpler to build and reproduce. The analyses did not include patients with missing data. Formal interaction tests were integrated in the Cox models to assess whether effects were different between subgroups. These interaction tests have low power and must be interpreted carefully. The Schoenfeld test and graphical evaluation of log (‐log survival time) versus log (time) plots were used to evaluate the proportional hazards assumptions.

Subgroup analyses for ECAD including tumor stage, MSI status, *BRAF* mutation status, tumor location, and tumor differentiation grade were conducted with the *a priori* knowledge that these covariates are associated with the protein expression of ECAD, and these analyses were therefore not corrected for multiple testing. The R functions wilcox.test and p.adjust were used to conduct two‐sided Wilcoxon rank‐sum tests with Benjamini–Hochberg false discovery rate (FDR) estimation adjustment [21] and with drug sensitivity scores as input. Differential ECAD protein expression between and among subgroups was evaluated using Wilcoxon rank‐sum test when comparing two groups and using Kruskal–Wallis test for comparing more than two groups. A *P*‐value < 0.05 (two‐tailed) was considered statistically significant.

## 3 Results

### 3.1 Staining patterns of colorectal cancer epithelial integrity markers

**Fig. 2:**
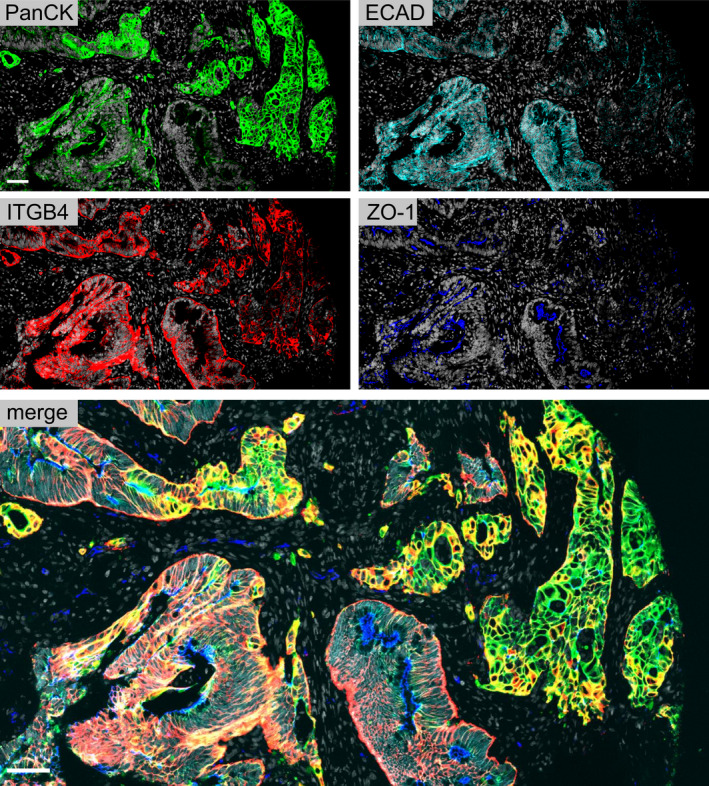
Staining patterns of epithelial integrity markers in primary colorectal cancer. Pan‐cytokeratin (PanCK) is shown in green; E‐cadherin (ECAD) is shown in cyan, integrin β4 (ITGB4) in red, and zona occludens 1 (ZO‐1) in blue. DAPI staining is shown in white. Scale bar, 50 µm.

The expressions of epithelial cell–cell adhesion and polarity markers were assessed in the Norwegian CRC series 1 (TMA1; *n* = 922, 689 evaluable cases, Table S2) by quantitative fluorescent multiplex immunohistochemistry of ZO‐1, ITGB4, ECAD, and cytokeratins (PanCK) (Fig. 2). The marker expression levels and patterns were analyzed both visually and by automated digital image analysis.

The visually inspected staining profiles of the epithelial markers showed expected patterns (Fig. 2; Fig. S2). The tight junction protein, ZO‐1, was localized to the apical surface of the intestinal epithelial cells in well‐differentiated carcinoma and to more random punctate foci in less polarized epithelium. In well‐differentiated and polarized epithelium, the hemidesmosome‐associated ITGB4 was localized to the basal membrane of the epithelial islands, whereas in higher grade tumors with invading structures, it had lost its membrane localization and appeared more in the cytoplasm. The adherence junction protein, ECAD, which was localized to the epithelial cell–cell contacts, showed the highest inter‐ and intratumoral variability from strong cell membrane staining to weaker intracellular punctate foci, and to almost total loss of staining. The intermediate filament proteins, cytokeratins, which physically connect cell–cell junctions and hemidesmosomes with the actin cytoskeleton and nuclei [22], appeared to inversely correlate with ECAD, showing stronger intensity in the epithelial areas with ECAD loss. By quantitative analysis, we found a weak positive correlation among ECAD, ITGB4, and ZO‐1 and a weak negative correlation between ECAD and cytokeratins (Fig. S3).

### 3.2 Clinical and molecular associations of epithelial integrity markers

We explored associations for the four markers to relevant clinical and molecular variables including stage, differentiation grade, location, microsatellite instability, and *BRAF* mutation status (Fig. S4A). ECAD, ITGB4, and ZO‐1 were inversely associated with tumor stage, while cytokeratins showed a positive association. As expected, ECAD showed a clear positive association with differentiation grade, while no significant differences were found for the three other markers. The distribution of ECAD showed a somewhat lower expression in right‐sided tumors as compared to left‐sided and rectal tumors as expected, whereas the opposite pattern was observed for cytokeratins. The distribution of ITGB4 showed higher expression in right‐sided tumors as compared to left‐sided and rectal tumors and ZO‐1 showed higher expression in rectal tumors as compared to left‐sided and right‐sided tumors. Microsatellite instability and *BRAF* mutation status were associated with low ECAD expression and high ITGB4 expression. None of the markers were associated with gender or age.

### 3.3 Association between markers of epithelial integrity and patient survival

The prognostic value of the continuous expression of each of the four markers was evaluated in univariable five‐year overall survival Cox models including all stages in the Norwegian series 1. We found that expression of ECAD (HR 0.78; 95% CI 0.70–0.86; *P* < 0.0001; *n* = 689), ITGB4 (HR 0.83; 95% CI 0.74–0.93; *P* = 0.0014; *n* = 689), and ZO‐1 (HR 0.89, 95% CI 0.79–1.00; *P* = 0.049, *n* = 689) were positively associated with survival, while cytokeratins showed a negative association (HR 1.18; 95% CI 1.05–1.32; *P* = 0.0041, *n* = 689). These relationships could also be illustrated by Kaplan–Meier analysis of the trichotomized variables (Fig. S5). A multivariable Cox model for stages I‐IV including all the four markers showed that ECAD, ITGB4, and cytokeratins carried independent prognostic information (Table S3A), but only ECAD was significant in full models including age, gender, stage, location, and microsatellite instability (stages I‐III and IV, Table 1 and stages I‐IV, Table S3B).

**Table 1:** Multivariable 5‐year overall survival (OS) Cox models of epithelial integrity markers. (A) Model including only E‐cadherin (ECAD), integrin β4 (ITGB4), zona occludens 1 (ZO‐1), and cytokeratins (PanCK). (B) Full model including relevant clinical and molecular variables. The continuous protein expression for all the markers were log_2_‐transformed and used as input in the models. Age was also included as a continuous variable in the models.

Variable (A)	Stage I–III (*n* = 578, 226 events)		Stage IV (*n* = 114, 107 events)	
	HR (CI)	*P* value	HR (CI)	*P* value
ECAD	0.83 (0.73–0.95)	0.0064	0.76 (0.64–0.91)	0.0021
ITGB4	0.91 (0.79–1.04)	0.16	0.94 (0.77–1.15)	0.56
ZO‐1	0.93 (0.81–1.06)	0.26	0.92 (0.74–1.14)	0.46
PanCK	1.20 (1.04–1.38)	0.010	1.00 (0.84–1.19)	0.99

Full Cox model (B)	Stage I–III (*n* = 506, 203 events)		Stage IV (*n* = 105, 98 events)	
	HR (CI)	*P* value	HR (CI)	*P* value
Age	1.05 (1.04–1.07)	< 0.0001	1.01 (1.00–1.03)	0.090
Gender				
Female	1	0.93	1	0.93
Male	1.18 (0.88–1.58)		0.98 (0.63–1.53)	
Stage				
I	1	< 0.0001		
II	1.49 (0.95–2.33)			
III	2.64 (1.68–4.15)			
Tumor location				
Right	1	0.66	1	0.32
Left	1.17 (0.83–1.65)		0.73 (0.45–1.18)	
Rectum	1.12 (0.75–1.66)		0.64 (0.32–1.28)	
Microsatellite instability				
MSS	1	0.036	1	0.66
MSI	0.58 (0.35–0.97)		1.22 (0.50–2.98)	
ECAD	0.81 (0.67–0.97)	0.023	0.73 (0.58–0.90)	0.004
ITGB4	0.99 (0.83–1.18)	0.92	0.99 (0.78–1.26)	0.95
ZO‐1	1.01 (0.87–1.18)	0.85	0.95 (0.74–1.22)	0.69
PanCK	1.13 (0.97–1.33)	0.12	0.84 (0.69–1.02)	0.081

### 3.4 Technical and clinical validation of ECAD as a prognostic biomarker in CRC

**Fig. 3:**
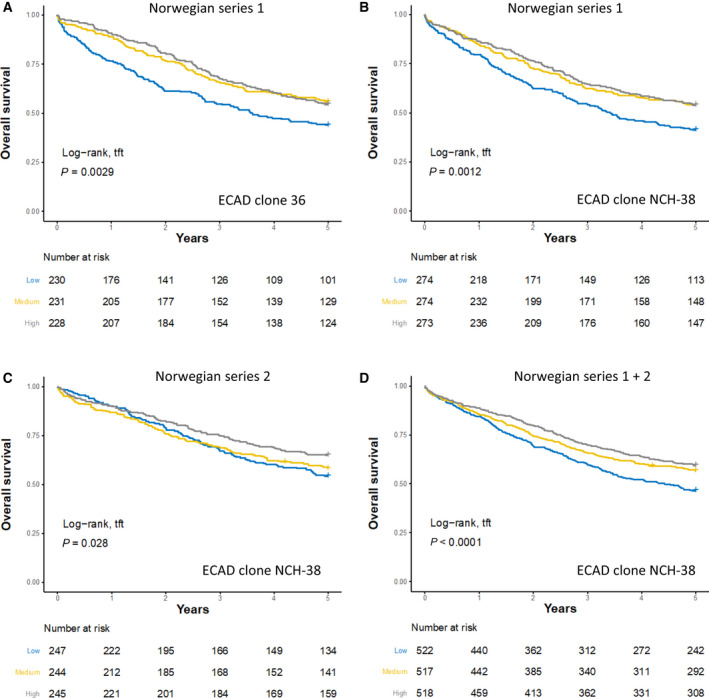
Technical and clinical validation of the prognostic value of E‐cadherin. Kaplan–Meier plots illustrating the prognostic associations for E‐cadherin (ECAD) using antibody clone 36 (A) and clone NCH‐38 (B) in Norwegian series 1. Prognostic validation was performed in Norwegian series 2 with clone NCH‐38 (C) and combined analyses for Norwegian series 1 and 2 are shown in (D). The continuous ECAD protein expression for each marker was trichotomized into three equal groups to facilitate Kaplan–Meier analysis and logrank test for trend (tft).

The prognostic value of ECAD as a continuous variable was technically validated in the Norwegian series 1 by different operators using a new monoclonal antibody (clone NCH‐38) and a separate mfIHC platform, employing different protocols for staining and digital image analysis (stages I‐IV 5‐year OS HR 0.84; 95% CI 0.76–0.92; *P* = 0.00015, *n* = 821, Fig. 3A,B).

Clinical validation using the NCH‐38 clone was performed in Norwegian series 2, showing highly comparable results (stages I‐IV, 5‐year OS HR 0.83; 95% CI 0.74–0.92; *P* = 0.00069, *n* = 736, Fig. 3C). Combined univariable Cox analysis of the Norwegian series 1 and 2 confirmed that ECAD protein expression is positively associated with prognosis in CRC (stages I‐IV, 5‐year OS HR 0.83; 95% CI 0.78–0.89; *P* < 0.0001; *n* = 1557, Fig. 3D). Multivariable Cox models in the combined series showed that the prognostic value of ECAD in stages I‐III and I‐IV (OS), and stages I‐III with complete resection (RFS) was independent of age, gender, stage, location, microsatellite instability, and *BRAF* mutation status (Table 2 and Table S4), as well as differentiation grade (Table S4). The prognostic effect of ECAD in stage IV was somewhat lower and not significant in a multivariable model including the same variables (Table 2).

**Table 2:** Multivariable Cox models of E‐cadherin in combined Norwegian series 1 and 2. Age and ECAD protein expression were included as continuous variables in the models. Log2‐transformation of ECAD was performed within each series before the values were standardized (*Z*‐score) and the series combined. ECAD, E‐cadherin; R0, complete resection/no residual tumor.

Variable	Stage I–III (OS) (*n* = 1158, 426 events)		Stage IV (OS) (*n* = 245, 215 events)		Stage I–III (R0, RFS) (*n* = 1108, 446 events)	
	HR (CI)	*P* value	HR (CI)	*P* value	HR (CI)	*P* value
Age	1.05 (1.04–1.06)	< 0.0001	1.03 (1.01–1.04)	< 0.0001	1.04 (1.03–1.05)	< 0.0001
Gender						
Female	1	0.11	1	0.07	1	0.1
Male	1.18 (0.96–1.43)		1.30 (0.98–1.74)		1.18 (0.97–1.43)	
Stage						
I	1	< 0.0001			1	< 0.0001
II	1.63 (1.21–2.21)				1.80 (1.34–2.41)	
III	2.79 (2.06–3.78)				2.98 (2.21–4.00)	
Tumor location						
Right	1	0.52	1	< 0.001	1	0.38
Left	1.15 (0.91–1.46)		0.73 (0.53–0.99)		1.17 (0.93–1.48)	
Rectum	1.07 (0.82–1.39)		0.40 (0.26–0.62)		1.15 (0.89–1.48)	
Microsatellite instability						
MSS	1	0.0076	1	0.73	1	0.004
MSI	0.56 (0.37–0.86)		0.90 (0.50–1.62)		0.55 (0.37–0.83)	
*BRAF*						
Wild‐type	1	0.14	1	0.14	1	0.17
Mutated	1.33 (0.91–1.96)		1.37 (0.91–2.09)		1.29 (0.90–1.87)	
ECAD	0.82 (0.74–0.91)	0.00026	0.92 (0.82–1.04)	0.18	0.87 (0.78–0.97)	0.0086

Associations between ECAD staining and stage, differentiation grade, location, microsatellite instability and *BRAF* mutation status were confirmed in the combined series (Fig. S4B), and we therefore speculated that ECAD might have particular prognostic value within these subgroups. This hypothesis was explored in univariable Cox models including all cases and in models restricted to patients with stages I‐III with complete resection (R0). Surprisingly, no significantly different prognostic effects were identified among the subgroups (Table 3). The prognostic effect appeared to be strongest in stage II and stage IV, but the differences were not statistically significant and lack a convincing functional and/or clinical rationale. No differential prognostic effects were either identified among age groups nor between genders. Thus, the prognostic value of ECAD appears to be largely independent of relevant clinical and molecular patient groups.

**Table 3:** Univariable Cox analyses of E‐cadherin in clinical and molecular subgroups. Norwegian series 1 and 2 were combined to explore potential prognostic subgroup effects of ECAD. The continuous ECAD protein expression was used as input in the analyses. Log2‐transformation of ECAD was performed within each series before the values were standardized (*Z*‐score) and the series combined. Formal interaction tests compare prognostic effects among the groups. CI, confidence interval; ECAD, E‐cadherin; HR, hazard ratio; ns, not significant; OS, overall survival; R0, complete resection; RFS, relapse‐free survival.

Variable	Stage I–IV (OS)			Stage I–III (R0, RFS)		
	HR (CI)	*P* value	*n*, events	HR (CI)	*P* value	*n*, events
All cases	0.83 (0.78–0.89)	< 0.0001	1557; 714	0.88 (0.80–0.96)	0.0039	1234; 496
Stage	*P*_interaction_ = ns			*P*_interaction_ = ns		
I	0.98 (0.76–1.27)	0.87	276; 63	0.99 (0.77–1.27)	0.91	275; 67
II	0.79 (0.69–0.91)	0.00076	605; 213	0.84 (0.74–0.96)	0.013	578; 227
III	0.91 (0.80–1.03)	0.14	409; 203	0.95 (0.83–1.08)	0.42	381; 202
IV	0.87 (0.78–0.98)	0.022	264; 233	–	–	–
Tumor location	*P*_interaction_ = ns			*P*_interaction_ = ns		
Right	0.81 (0.73–0.91)	0.0003	637; 300	0.88 (0.76–1.02)	0.08	503; 196
Left	0.83 (0.74–0.93)	0.0017	494; 247	0.83 (0.71–0.96)	0.015	375; 163
Rectum	0.91 (0.62–0.88)	0.2	391; 151	0.95 (0.80–1.12)	0.54	327; 126
Tumor grade^a^	*P*_interaction_ = ns			*P*_interaction_ = ns		
High	0.89 (0.66–1.20)	0.44	108; 41	1.01 (0.72–1.42)	0.96	96; 36
Moderate	0.85 (0.78–0.92)	0.00016	1199; 531	0.91 (0.82–1.01)	0.086	969; 385
Low	0.91 (0.76–1.08)	0.28	204; 121	0.80 (0.61–1.04)	0.097	135; 61
Microsatellite instability	*P*_interaction_ = ns			*P*_interaction_ = ns		
MSI	0.79 (0.63–0.99)	0.043	229; 85	0.80 (0.62–1.03)	0.088	203; 67
MSS	0.81 (0.75–0.88)	< 0.0001	1248; 590	0.85 (0.76–0.94)	0.0017	967; 403
*BRAF*	*P*_interaction_ = ns			*P*_interaction_ = ns		
Wild‐type	0.84 (0.78–0.92)	0.000089	1244; 564	0.88 (0.79–0.98)	0.018	977; 393
Mutated	0.74 (0.62–0.88)	0.00092	235; 114	0.77 (0.60–0.98)	0.037	190; 77

^a^ Tumor differentiation grade.

To indirectly evaluate whether ECAD expression is associated with benefit from adjuvant chemotherapy, we compared 5‐year RFS (R0) Kaplan–Meier models of chemotherapy versus no chemotherapy as a function of ECAD expression divided into three equal groups. The survival differences were similar among the groups and formal interaction tests did not identify any significant differences (Fig. S6). Similar results were obtained using overall survival as clinical endpoint, by evaluating two or five equal ECAD staining groups, and when using continuous data as input.

### 3.5 Pharmacoproteomic associations of ECAD expression *in vitro*

**Fig. 4:**
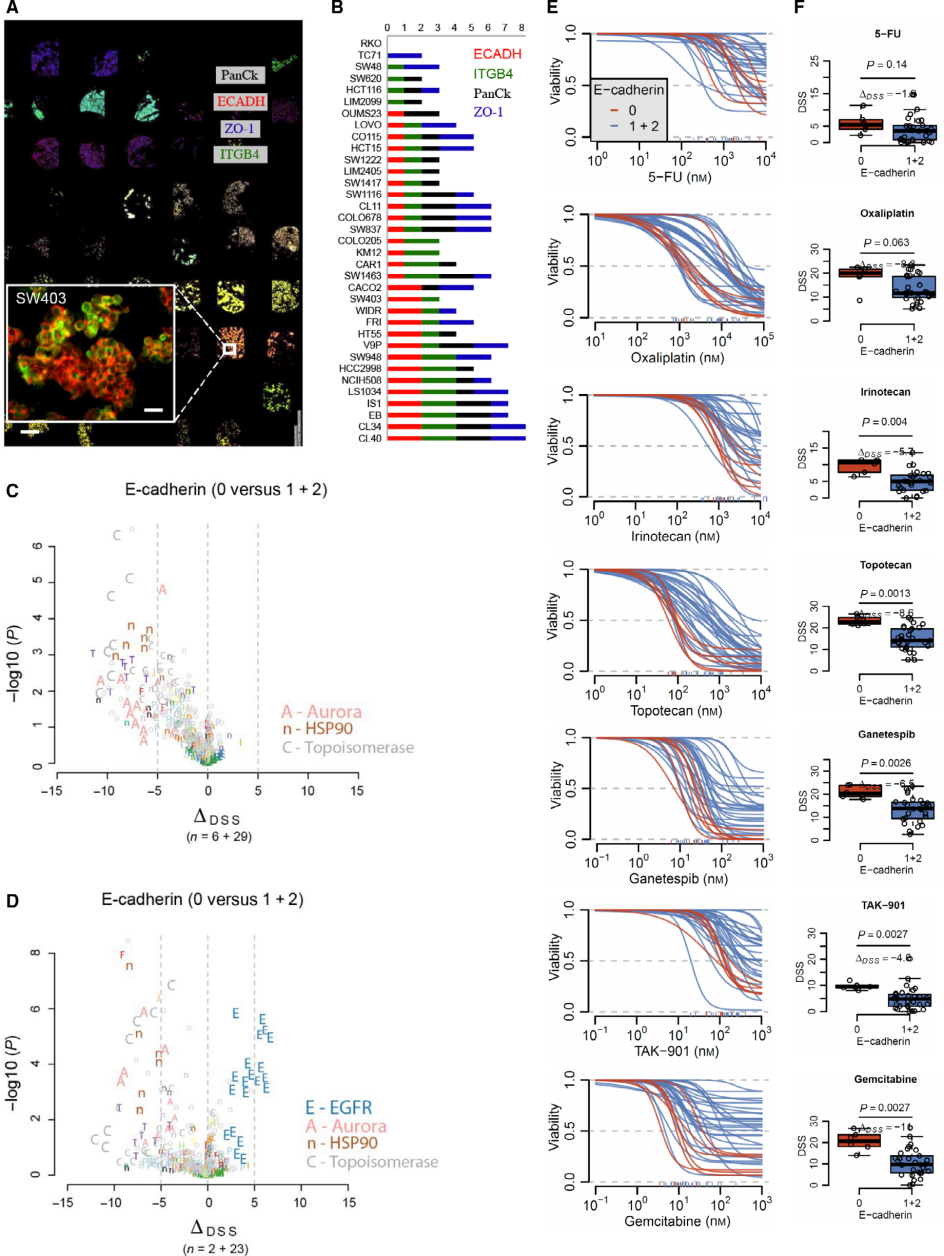
Pharmacoproteomic evaluation of epithelial integrity markers *in vitro*. (A) Cell line paraffin array with epithelial marker staining of 35 unique colorectal cancer cell lines. Scale bars, 25 µm (magnified area) and 500 µm. (B) Epithelial marker expression in cell lines with 3‐tier scoring (0 = null, 1 = medium, 2 = high). (C) Volcano plots showing differences in drug sensitivity between cell lines with loss of ECAD and cell lines with medium or high ECAD expression including all cell lines and (D) only microsatellite stable cell lines (please note the low number of cell lines with loss of ECAD when interpreting the figure). (E) Curves showing estimated dose–response models for all cell lines for seven selected drugs. Each curve represents a cell line and is colored according to ECAD status (red = ECAD null). Concentrations are in nanoMolars (nm) with dose‐window defined by screening range. (F) Corresponding boxplots illustrating difference in cell line drug sensitivity scores (DSS, proportional to area over the curve) for the same seven drugs. *P*‐values are from Wilcoxon tests and delta DSS is the median difference. All 35 cell lines were screened according to a standardized protocol and the data presented are based on one high‐throughput drug screen per cell line. DSS, drug sensitivity score; ECAD, E‐cadherin; ITGB4, integrin β4; PanCK, pan‐cytokeratin; ZO‐1, zona occludens 1.

Potential associations between ECAD expression and drug sensitivity in preclinical models were explored by staining a cell line microarray including 35 unique CRC cell lines (Fig. 4A,B) annotated with 293 approved and investigational drugs (DSS_max_ > 10). The cell lines showed a trimodal distribution of ECAD where six cell lines had a particularly low expression, and these cell lines were compared against the rest in differential sensitivity analyses including all drugs. We found that cell lines with loss of ECAD expression were significantly more sensitive to topoisomerase, aurora, and HSP90 inhibitors (Fig. 4C,D, Table S5), here illustrated by comparing dose–response curves and drug sensitivity scores for irinotecan, topotecan, TAK‐901, and ganetespib, respectively (Fig. 4E,F). This observation appeared to be independent of microsatellite instability (Fig. 4D and Table S6). MSS cell lines with moderate to high ECAD expression were more sensitive to EGFR inhibition (Fig. 4D and Table S6). We observed no strong association between ECAD expression and sensitivity to 5‐FU, but loss of ECAD expression was associated with sensitivity to oxaliplatin and gemcitabine (Fig. 4E,F).

## 4 DISCUSSION

During the early phases of the metastatic process, carcinoma cells often undergo EMT, where adherence junctions (ECAD), apical tight junctions (ZO‐1), and basolateral hemidesmosomes (ITGB4) are disassembled [2, 3, 23]. Although the functional roles of these epithelial integrity proteins have been extensively studied in cancer, their relative potential as prognostic markers in CRC has not been thoroughly investigated in large representative patient series. By use of the mfIHC technology, we could analyze the continuous expressions with a large linear dynamic range, of the selected markers on the same tissue section. We found that ECAD expression was most strongly associated with patient survival among the four markers. Although ITGB4 and PanCK both showed independent prognostic information from ECAD, the only marker with independent prognostic value in multivariable models was ECAD. The current data confirm previous studies applying conventional chromogenic DAB staining and visual scoring assessing ECAD as a prognostic marker in CRC [24, 25].

In this study, we observed inverse association of ECAD and PanCK expression, and they were also inversely associated with disease progression and patient survival in CRC. The loss or decrease in the expression of epithelial ECAD has been linked with tumor budding and more aggressive disease in CRC [24, 26, 27]. However, the association of cytokeratins with CRC progression and survival is more complex: Colonocyte differentiation associated cytokeratins, CK20 and CK8, may be partially lost during EMT or invasion, but other cytokeratins, such as CK7, CK18, and CK19, have been associated with increased tumor burden, tumor budding, invasion, or poor survival [22]. Indeed, the pan‐cytokeratin antibody mix we have used and named as PanCK in this study (clones C‐11, AE1, AE3) detects multiple types of cytokeratins, and individual cytokeratins may have a tissue context‐dependent expression profile and role, thus also showing strong interpatient and intrapatient variations. For example, CK23 is more pronounced in MSS tumors and is associated with more aggressive CRC [28, 29]. Given that pan‐cytokeratin IHC, typically with clones AE1/AE3 is robustly used to detect aggressive CRC associated tumor budding [30], suggests that cytokeratins are not readily lost during invasion process. Consistent with this, EMT has been considered more like a partial EMT in CRC [31], where in fact double‐positive cells for pan‐cytokeratin and “mesenchymal” vimentin mark more aggressive tumor cells [32]. We have also previously observed in stage II colon patient tumor samples that while ECAD expression levels were lower in tumor invasive border and tumor buds than in tumor center, cytokeratin expression levels were higher in the tumor invasive border and in tumor buds [26]. Further analyses are warranted to elaborate which individual cytokeratins are over‐ and underrepresented in tumor cells with a loss of ECAD.

Mechanistically, ECAD association with unfavorable survival in CRC could be linked with the onset of invasion (EMT) and induction of cancer stem cell phenotype (CSCs) at least through Wnt‐beta‐catenin pathway. Deletion of APC or activating mutation of beta‐catenin is an early event in colorectal carcinomas (> 75%), pointing out that these cancers largely depend on Wnt signaling activation [33]. Experimental studies have shown that aberrant ECAD leads to disruption of ECAD‐beta‐catenin cell–cell junctional complex, activation of Wnt signaling, translocation of cell junctional beta‐catenin to nucleus, and induction of cancer stem cells (CSCs) [34], which are considered drivers of tumor progression and metastasis of CRC [35]. Cytokeratins, in turn, are intermediate filaments forming physical links with the actin cytoskeleton and extracellular matrix through integrins and other cell membrane proteins [22]. Knockout and overexpression studies have shown that cytokeratins may dramatically influence cell shape, adhesion, migration, invasion, as well as metastatic properties of tumor cells [22].

Despite the high biomarker discovery rate, very few biomarkers are robustly validated and even fewer are implemented in the clinic [8]. We have here optimized a robust protein detection protocol combined with a simple algorithm for digital image analysis of the total protein expression in the epithelial cancer tissue. The subsequent biomarker analyses were in line with the REMARK guidelines to ensure that the results are reproducible and comparable with results from independent laboratories. We have also attempted to follow best practices for mfIHC staining and validation [36]. A particular strength with our study is the confirmed independent prognostic value of ECAD applying two different monoclonal antibodies and two different mfIHC platforms. We found ECAD to be a robust prognostic marker, independent of the MSI status, as well as of other relevant clinical and molecular subgroups. Finally, the fact that the biological function of ECAD is strongly associated with cancer progression and metastasis is a preferred characteristic of a good biomarker. These results together substantiate the potential clinical value of ECAD.

We had no *a priori* hypotheses for which patient subgroups the different markers would show particular prognostic effects. Hence, the conducted subgroup analyses were exploratory. However, ECAD showed strongest prognostic effect in stage II (Table 3) suggesting that ECAD IHC staining of central tumor after curative surgery might be a clinically useful biomarker to help identify stage II CRC patients in need for more intense follow‐up. However, this finding needs to be externally validated. Further studies are also needed to determine the most appropriate method for measuring ECAD protein expression in a clinical setting, but this study and our previous study using chromogenic staining and visual scoring of TMAs [24] suggest that ECAD has robust prognostic value across technological platforms and with both fluorescent and chromogenic detection methods, as well as with both visual and digital image analysis. We propose that the prognostic value of ECAD should be externally validated for use in multivariable prediction models as a continuous variable according to the TRIPOD guidelines for risk stratification [37]. Furthermore, all the tissue cores were from the central tumor region; hence, the ECAD expression is likely more stable as compared to the invasive front and is easier to measure in a reproducible manner. However, validation studies using whole tissue sections or TMAs with cores from multiple sites of the tumor are needed to determine the clinical utility of ECAD as a biomarker. Although individual TMA CRC tissue cores show more variability than whole tissue sections, the strong association between low ECAD expression and poor prognosis is robust due to the large number of patients included from two independent consecutive patient cohorts, and this association is likely to become even stronger when analyzing a larger part of the tumor.

Our analyses did not show any apparent associations of ECAD protein expression and survival among stage III related to whether the patients have received adjuvant chemotherapy including almost 400 patients, which should be sufficient to discover clinically relevant signals. But the analysis is limited by being retrospective and the included patients are not therapy‐randomized, which is ultimately necessary to answer this question properly. Patients offered adjuvant chemotherapy have in general a better performance status and are younger than those who did not receive adjuvant chemotherapy. However, there was still no association when the predictive analyses were restricted to colon cancer patients below 75 years of age.

Subsequent exploratory analyses in a relatively large panel of CRC cell lines did however identify an interesting association between loss of ECAD and response to topoisomerase, aurora, and HSP90 inhibitors. The first might have clinical implications for evaluation of response to irinotecan, which is used in standard of care combination chemotherapy.

The link between ECAD and aurora inhibitors is not novel, as earlier work revealed that the combination of aurora inhibitors and ECAD deficiency is highly efficient in killing breast cancer cells [38, 39]. However, to our knowledge, this connection has not been previously reported in CRC. We have previously, based on partly overlapping data, reported associations between gene expression subtypes and HSP90 inhibitor sensitivity [16]. The congruity is likely attributable to biological connections between gene expression subtypes, differentiation, and ECAD expression.

EGFR‐targeted therapy is commonly used in patients with *RAS* wild‐type metastatic CRC, but acquired resistance develops rapidly in most patients. Results from multiple studies in lung cancer suggest that epithelial phenotype with increased *ECAD* gene (*CDH1*) or protein expression, as opposed to mesenchymal or EMT phenotype, is associated with increased sensitivity to EGFR inhibitors [40, 41, 42, 43, 44, 45]. Moreover, functional experiments have shown that EGFR inhibitor sensitivity is directly related to increased ECAD expression in colon cancer [46] and in lung cancer [47, 48]. Furthermore, colon cancer patients who responded (*n* = 19) to antibody‐based EGFR inhibition therapy (cetuximab) had significantly higher cancer tissue expression of ECAD (IHC scoring) than patients not responding (*n* = 17) [49] to treatment. Thus, our results of increased EGFR inhibitor sensitivity of CRC cells with MSS phenotype and higher ECAD expression are consistent with earlier studies and further validate this association. Although the results from the pharmacoproteomic analyses are intriguing and supported by independent reports, they are limited by the relatively low number of cell lines with loss of ECAD.

The pharmacoproteomic associations identified warrant further studies to address limitations inherent to our approach. For instance, to what extent does the expression of epithelial markers change under different growth conditions (e.g., confluency versus exponential growth). Relatedly, although colorectal cancer cell lines are representative disease models [50, 51], these potential pharmacoproteomic relationships should be investigated further in induction and knockout experiments as well as using more advanced cancer models, such as patient‐derived organoids and tumor xenografts in mice.

In the present study, we investigated whether proteins implicated in maintaining epithelial integrity are associated with patient prognosis and cancer cell drug sensitivity in colorectal carcinoma. We used mfIHC to simultaneously detect adherence junction protein ECAD, hemidesmosomal adhesion protein ITGB4, tight junction protein ZO‐1, and intermediate filament proteins, cytokeratins, both in patient samples and in cell lines. We found and validated ECAD as a robust and independent predictor of patient survival, but not as predictor of response to adjuvant chemotherapy in CRC. Our preclinical drug screen results validated the association of ECAD expression and EGFR inhibitor sensitivity. We discovered that low ECAD expression is associated with sensitivity to topoisomerase, aurora, and HSP90 inhibitors in CRC models.

## 5 CONCLUSION

Reproducibility of biomarker results across technical and analytical platforms, and across laboratories shows that fluorescent immunohistochemical analysis of ECAD can be robustly standardized in diagnostic laboratories. ECAD can be a clinically useful biomarker to guide management of patients with CRC.

## Conflict of interest

The authors declare no conflict of interest.

## Author contributions

JB and TP conceived and designed the study. OB, MA, KV, and TP performed the discovery mfIHC experiments on the Norwegian series 1 TMA and the cell line microarrays, and JB and MB performed the technical and clinical validation mfIHC experiments of ECAD on the Norwegian series 1 and 2. JB, PWE, and TP performed and analyzed the pharmacoproteomic experiments. JB, PWE, CHB, OB, AS, MA, KV, MB, MGG, OK, AN, RAL, and TP acquired biological and/or clinical data. JB, PWE, OB, CHB, MG, AN, RAL, and TP analyzed and interpreted the data. JB drafted the manuscript, and all authors were involved in revision of the manuscript and have read and approved the final version. JB, RAL, and TP supervised the study.

### Peer review

The peer review history for this article is available at https://publons.com/publon/10.1002/1878‐0261.13159.

## Supporting information


**Fig. S1**. Kaplan‐Meier plots illustrating prognostic associations for tumor differentiation grade in Norwegian series 1 and 2 (stages I‐IV, OS).
**Fig. S2**. Example image of mfIHC staining with a magnification.
**Fig. S3**. Correlation among epithelial integrity markers.
**Fig. S4**. Clinical and molecular associations for epithelial integrity markers.
**Fig. S5**. Prognostic associations for epithelial integrity markers in primary colorectal cancer.
**Fig. S6**. Predictive evaluation of ECAD for association with adjuvant chemotherapy.
**Table S1**. REMARK Checklist.
**Table S2**. Patient characteristics for Norwegian series 1 and 2.
**Table S3**. Multivariable Cox models including the epithelial integrity markers (stages I‐IV, OS).
**Table S4**. Multivariable Cox model of ECAD in Norwegian series 1 and 2 (stages I‐IV, OS).
**Table S5**. Top 25 drugs based on differential drug sensitivity analysis between cell lines with loss of ECAD and medium to high expression of ECAD (MSS and MSI cell lines included).
**Table S6**. Top 25 drugs based on differential drug sensitivity analysis between cell lines with loss of ECAD and medium to high expression of ECAD (only MSS cell lines included).

## Data Availability

The data that support the findings of this study are available from the corresponding authors teijo.pellinen@helsinki.fi and jarlebruun@gmail.com upon reasonable request.
