# Supplementary material for: E‐cadherin is a robust prognostic biomarker in colorectal cancer and low expression is associated with sensitivity to inhibitors of topoisomerase, aurora, and HSP90 in preclinical models
Source: Mol Oncol. 2021 Dec 26;16(12):2312–29. doi: 10.1002/1878-0261.13159 (PMC9208074; doi:10.1002/1878-0261.13159)
Supplement: Supplementary file 1 — Fig. S1. Kaplan‐Meier plots illustrating prognostic associations for tumor differentiation grade in Norwegian series 1 and 2 (stages I‐IV, OS). Fig. S2. Example image of mfIHC staining with a magnification. Fig. S3. Correlation among epithelial integrity markers. Fig. S4. Clinical and molecular associations for epithelial integrity markers. Fig. S5. Prognostic associations for epithelial integrity markers in primary colorectal cancer. Fig. S6. Predictive evaluation of ECAD for association with adjuvant chemotherapy. Table S1. REMARK Checklist. Table S2. Patient characteristics for Norwegian series 1 and 2. Table S3. Multivariable Cox models including the epithelial integrity markers (stages I‐IV, OS). Table S4. Multivariable Cox model of ECAD in Norwegian series 1 and 2 (stages I‐IV, OS). Table S5. Top 25 drugs based on differential drug sensitivity analysis between cell lines with loss of ECAD and medium to high expression of ECAD (MSS and MSI cell lines included). Table S6. Top 25 drugs based on differential drug sensitivity analysis between cell lines with loss of ECAD and medium to high expression of ECAD (only MSS cell lines included). [file MOL2-16-2312-s001.docx]

# Supporting information

E-cadherin is a robust prognostic biomarker in colorectal cancer and low expression is associated with sensitivity to inhibitors of topoisomerase, aurora and HSP90 in preclinical models

| **Item to be reported** | | **Page no.** |
| --- | --- | --- |
| **INTRODUCTION** | |  |
| 1 | State the marker examined, the study objectives, and any pre-specified hypotheses. | 3,5 |
| **MATERIALS AND METHODS** | |  |
| *Patients* | |  |
| 2 | Describe the characteristics (e.g., disease stage or co-morbidities) of the study patients, including their source and inclusion and exclusion criteria. | 6,7 |
| 3 | Describe treatments received and how chosen (e.g., randomized or rule-based). | 6 |
| *Specimen characteristics* | |  |
| 4 | Describe type of biological material used (including control samples) and methods of preservation and storage. | 6,7 |
| *Assay methods* | |  |
| 5 | Specify the assay method used and provide (or reference) a detailed protocol, including specific reagents or kits used, quality control procedures, reproducibility assessments, quantitation methods, and scoring and reporting protocols. Specify whether and how assays were performed blinded to the study endpoint. | 7-12 |
| *Study design* | |  |
| 6 | State the method of case selection, including whether prospective or retrospective and whether stratification or matching (e.g., by stage of disease or age) was used. Specify the time period from which cases were taken, the end of the follow-up period, and the median follow-up time. | 6-7 |
| 7 | Precisely define all clinical endpoints examined. | 10,11 |
| 8 | List all candidate variables initially examined or considered for inclusion in models. | 10,11 |
| 9 | Give rationale for sample size; if the study was designed to detect a specified effect size, give the target power and effect size. | 6-7 |
| *Statistical analysis methods* | |  |
| 10 | Specify all statistical methods, including details of any variable selection procedures and other model-building issues, how model assumptions were verified, and how missing data were handled. | 10,12 |
| 11 | Clarify how marker values were handled in the analyses; if relevant, describe methods used for cutpoint determination. | 10,11 |
| **RESULTS** | |  |
| *Data* | |  |
| 12 | Describe the flow of patients through the study, including the number of patients included in each stage of the analysis (a diagram may be helpful) and reasons for dropout. Specifically, both overall and for each subgroup extensively examined report the numbers of patients and the number of events. | Figure1,  Figure3,  Table 1-3 |
| 13 | Report distributions of basic demographic characteristics (at least age and sex), standard (disease-specific) prognostic variables, and tumor marker, including numbers of missing values. | Table S2 |
| *Analysis and presentation* | |  |
| 14 | Show the relation of the marker to standard prognostic variables. | Table S2 |
| 15 | Present univariable analyses showing the relation between the marker and outcome, with the estimated effect (e.g., hazard ratio and survival probability). Preferably provide similar analyses for all other variables being analyzed. For the effect of a tumor marker on a time-to-event outcome, a Kaplan-Meier plot is recommended. | 14 and Figure S5 |
| 16 | For key multivariable analyses, report estimated effects (e.g., hazard ratio) with confidence intervals for the marker and, at least for the final model, all other variables in the model. | Table 1, 2, S3 and S4 |
| 17 | Among reported results, provide estimated effects with confidence intervals from an analysis in which the marker and standard prognostic variables are included, regardless of their statistical significance. | Table 1, 2, S3 and S4 |
| 18 | If done, report results of further investigations, such as checking assumptions, sensitivity analyses, and internal validation. | 10,11 |
| **DISCUSSION** | |  |
| 19 | Interpret the results in the context of the pre-specified hypotheses and other relevant studies; include a discussion of limitations of the study. | 16-19 |
| 20 | Discuss implications for future research and clinical value. | 16-19 |

#### Table S1. REMARK Checklist

#### Table S2. Patient characteristics for Norwegian series 1 and 2

^a^*P*-value for association with E-cadherin protein expression

^b^Excluded from association analyses with E-cadherin protein expression

^c^Cases with mucinous tumors were pooled with tumors with low differentiation

Abbreviations: ns, not significant; ND, not determined; R0, complete resection/no residual tumor; R1, microscopic residual cancer at the resection margin; R2, macroscopic or radiological evidence of residual cancer, locally or distant.

#### Table S3| Multivariable Cox models including the epithelial integrity markers (stages I-IV, OS)

Abbreviations: ECAD, E-cadherin; ITGB4, integrin beta-4; zona occludens-1; PanCK, pan-cytokeratins; MSS, microsatellite stable; MSI, microsatellite instable; OS, overall survival. Green color indicates the adjusted values for ECAD when tumor differentiation grade is included in the multivariable model shown above.

#### Table S4| Multivariable Cox model of ECAD in Norwegian series 1 and 2 (stages I-IV, OS)

Abbreviations: ECAD, E-cadherin; MSS, microsatellite stable; MSI, microsatellite instable; OS, overall survival. Green color indicates the adjusted values for ECAD when tumor differentiation grade is included in the multivariable model shown above.

#### Table S5 | Top 25 drugs based on differential drug sensitivity analysis between cell lines with loss of E‑cadherin and medium to high expression of ECAD (MSS and MSI cell lines included).

Abbreviations: diff.DSS, differential drug sensitivity score; ECAD, E-cadherin; FDR, false discovery rate; moa, mechanism of action; ph, phase.

#### Table S6 | Top 25 drugs based on differential drug sensitivity analysis between cell lines with loss of E‑cadherin and medium to high expression of ECAD (only MSS cell lines included)

Abbreviations: diff.DSS, differential drug sensitivity score; ECAD, E-cadherin; FDR, false discovery rate; moa, mechanism of action; ph, phase


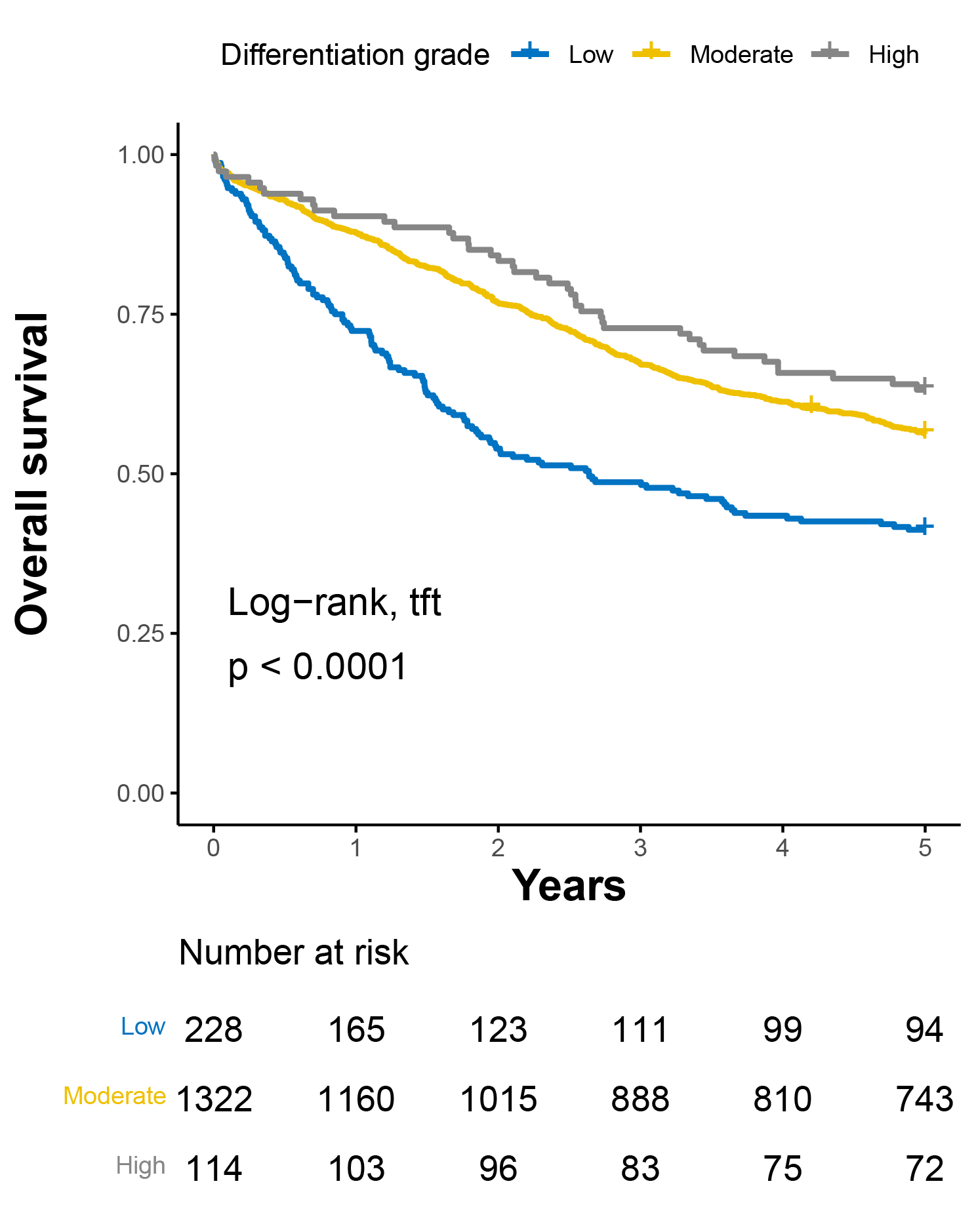


Figure S1 | Kaplan-Meier plots illustrating prognostic associations for tumor differentiation grade in Norwegian series 1 and 2, colorectal cancer stages I-IV. Abbreviations: tft, test for trend.


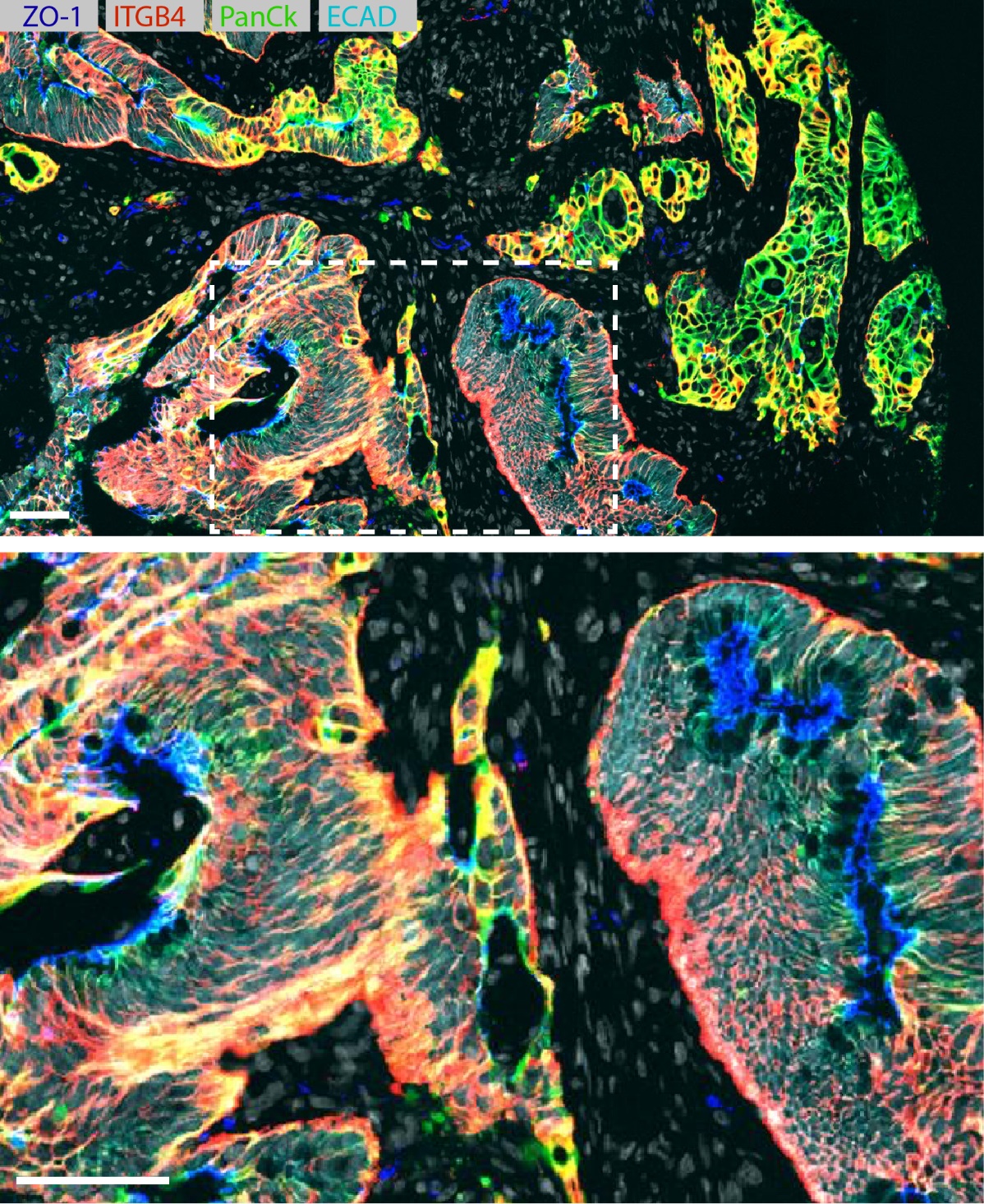


Figure S2 | Example image of mfIHC staining with a magnification. Scale bar, 50 µm.

Abbreviations: ZO-1, zona occludens-1; ITGB4, integrin beta-4; ECAD, E-cadherin; PanCK, pan‑cytokeratins

#### Figure S3 | Correlation among epithelial integrity markers

Abbreviations: ZO-1, zona occludens-1; ITGB4, integrin beta-4; ECAD, E-cadherin; PanCK, pan‑cytokeratins


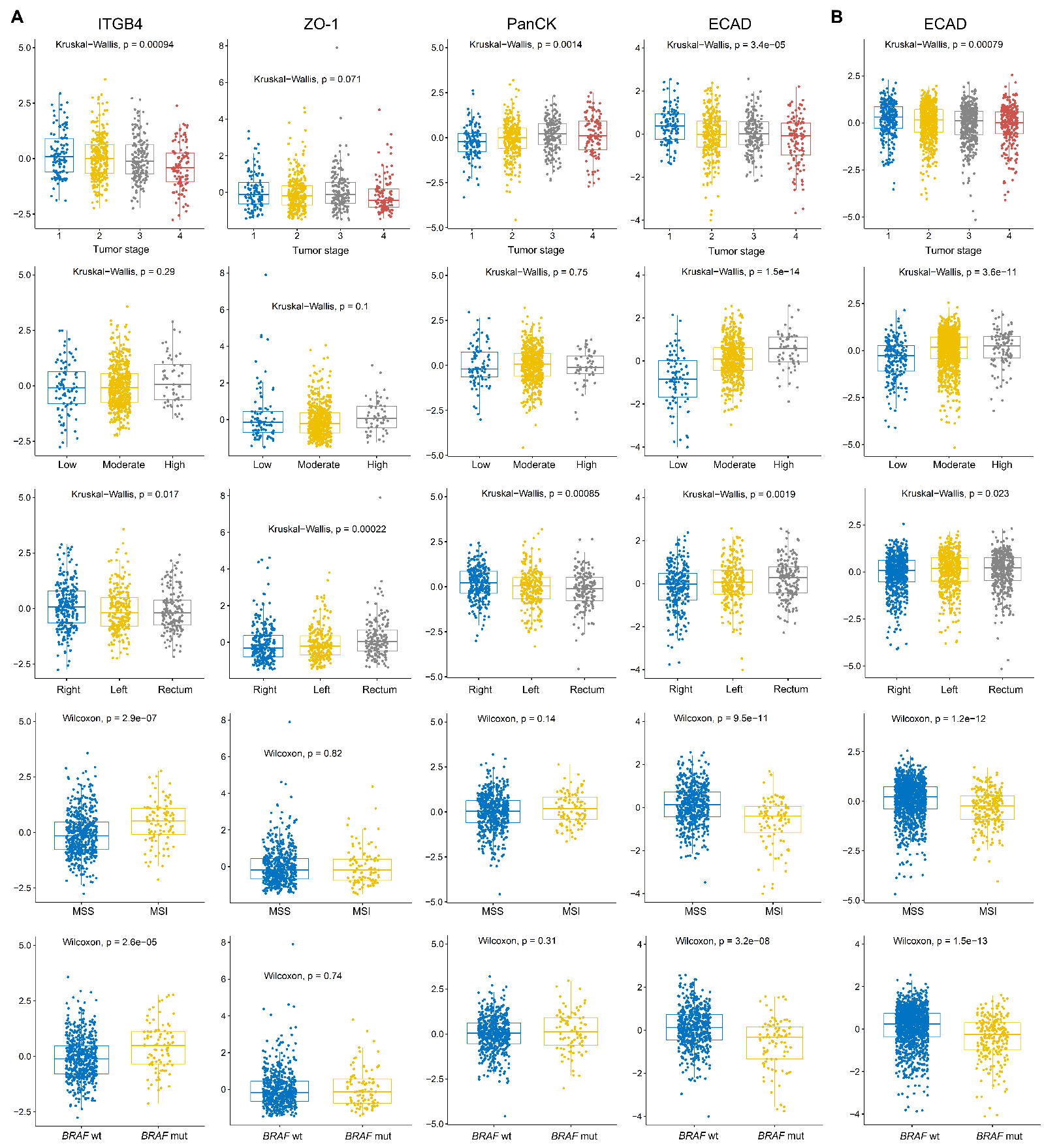


#### Figure S4 | Clinical and molecular associations for epithelial integrity markers

Clinical and molecular associations for ECAD in the Norwegian series 1 (A) and in the combined Norwegian series (B). Rows from top to bottom: tumor stage, differentiation grade, tumor location, MMR status, *BRAF* mutation status. Abbreviations: ZO-1, zona occludens-1; ITGB4, integrin beta-4; ECAD, E-cadherin; PanCK, pan‑cytokeratins.


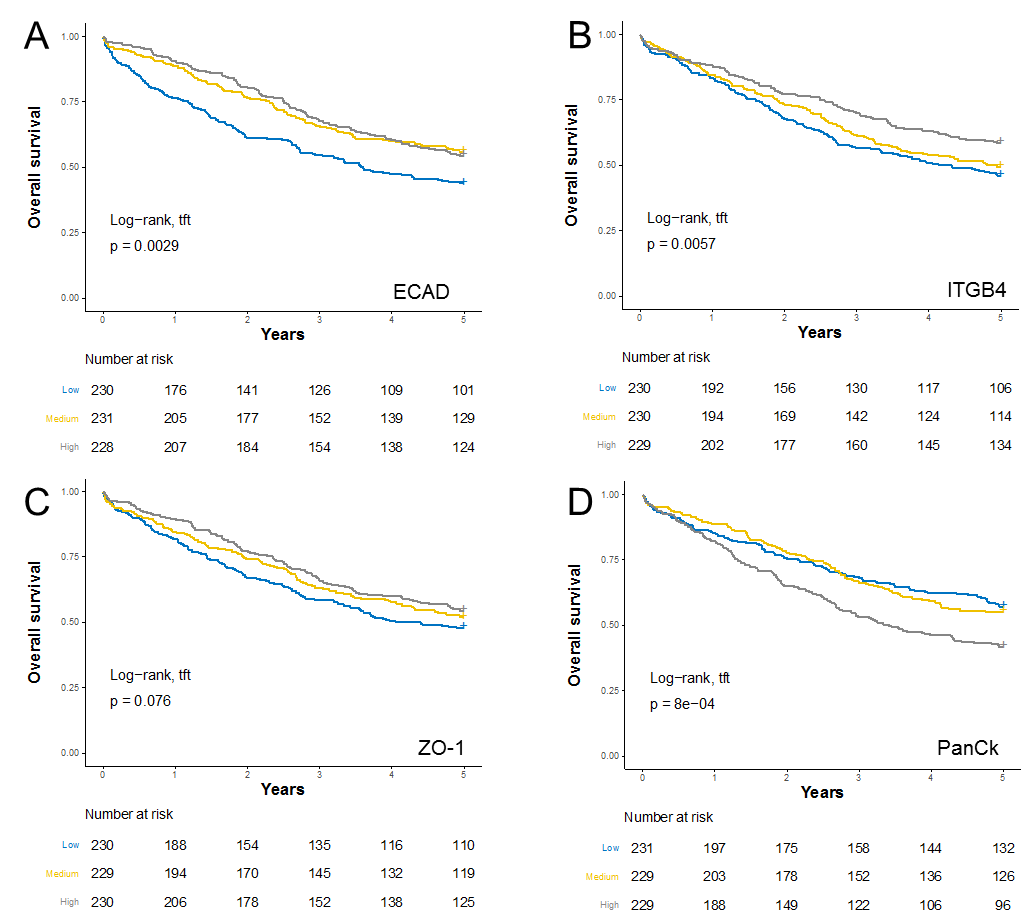


#### Figure S5| Prognostic associations for epithelial integrity markers in primary colorectal cancer

Kaplan-Meier plots illustrating the prognostic associations for E-cadherin (ECAD, A), integrin β4 (ITGB4, B), zona occludens-1 (ZO-1, C) and cytokeratins (PanCK, D) in Norwegian series 1. The continuous protein expression for each marker was trichotomized into three equal groups to facilitate Kaplan-Meier analysis and logrank test for trend (tft).


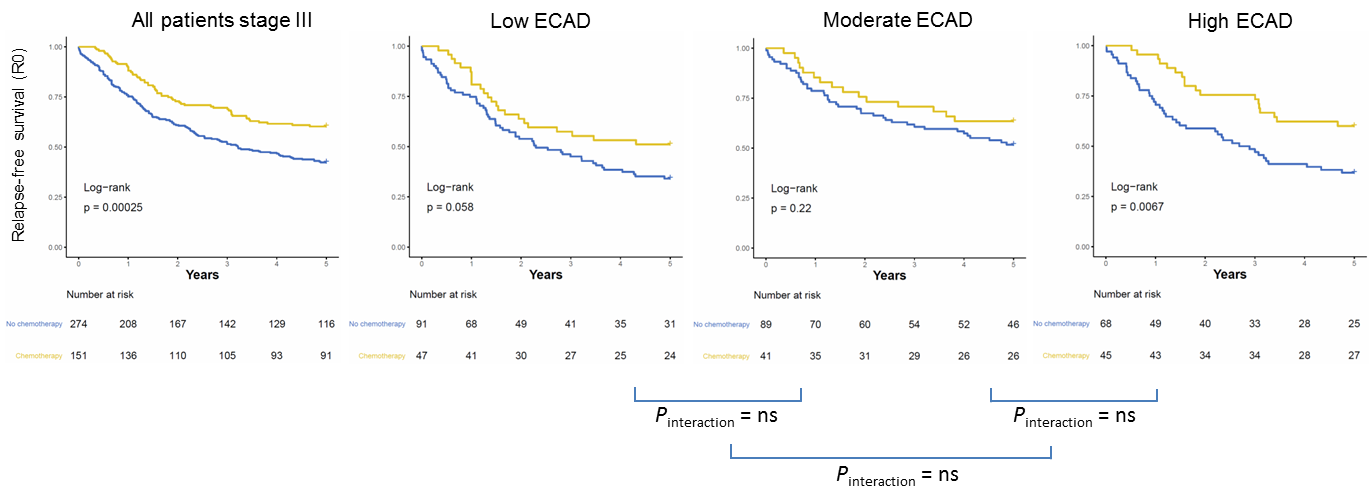


#### Figure S6 | Predictive evaluation of ECAD for association with adjuvant chemotherapy

ECAD protein expression was trichotomized into three equal groups and Kaplan-Meier analysis and logrank tests were performed for each group. Formal interaction tests compare prognostic effects among the three groups with low, moderate, or high ECAD expression. Abbreviations: R0, complete resection; ns, not significant; ECAD, E-cadherin.
